# Diagnostic Approach to Genetic Obesity in Children: From Pathophysiology to Precision Medicine—A Narrative Review

**DOI:** 10.3390/medicina62101850

**Published:** 2026-09-24

**Authors:** Cristina Partenope, Ivana Rabbone, Roberta Pajno

**Affiliations:** 1 Division of Pediatrics, Department of Health Sciences, University of Piemonte Orientale, Ospedale Maggiore della Carità, 28100 Novara, Italy; ivana.rabbone@uniupo.it; 2 Department of Pediatrics, IRCCS San Raffaele Hospital, 20132 Milan, Italy; pajno.roberta@hsr.it

**Keywords:** childhood obesity, monogenic obesity, syndromic obesity, leptin–melanocortin pathway, genetic testing, precision medicine, polygenic risk score

## Abstract

Childhood obesity is a major global health concern with a strong heritable component. While most cases are polygenic and environmentally driven, a subset of children present with rare genetic forms of obesity, including syndromic and monogenic disorders. Over the past three decades, advances in gene discovery—from candidate gene studies to genome-wide association studies (GWAS) and next-generation sequencing (NGS)—have significantly improved our understanding of the biological pathways regulating appetite and energy balance, particularly the leptin–melanocortin pathway. Early identification of genetic obesity is clinically relevant, as it allows for tailored management, targeted pharmacological interventions, genetic counseling, and a reduction in psychosocial stigma. However, distinguishing rare genetic obesity from common polygenic obesity remains challenging in clinical practice. This narrative review summarizes the current knowledge on the genetic architecture of pediatric obesity, explores the transition from gene discovery to precision medicine, discusses available diagnostic algorithms and genetic testing strategies, and proposes an updated diagnostic flowchart. In a more clinically useful model, genetic obesity is not defined solely by the presence or absence of a pathogenic variant, but as a continuum in which phenotype, genetic variants, age, and environmental exposure interact and define risk prediction and therapeutic strategies, especially in the pediatric population.

## 1. Introduction

Obesity is a complex pathological condition defined as abnormal or excessive body fat and histologically characterized by the presence of hyperplastic and hypertrophic adipocytes [1].

In clinical practice, the definition of obesity is based on the assessment of the body mass index (BMI), calculated as the body weight expressed in kilograms divided by the height in square meters. Differently from adulthood, age- and sex-specific reference percentile curves are used for the diagnosis of overweight and obesity in pediatrics [2]. In children under 2 years of age, the diagnosis is based on the weight/length ratio according to the 2006 World Health Organization (WHO) reference curves: a value between the 97th and 99th percentile is classified as overweight, while >99th as obesity. The BMI cut-off for obesity diagnosis is the 99th centile in children aged 2–5 years and 97th in children aged 5–18 years [3]. Besides BMI (which does not reflect body fat deposition), waist circumference and skin-fold thickness are the most useful noninvasive clinical measures to assess overall adiposity. A waist-to-height ratio > 0.5 or waist circumference > 90th percentile is associated with increased cardiovascular risk [4].

The incidence of severe obesity in the pediatric population has grown worldwide, with significant financial and social burden. The WHO has reported that approximately 39 million children under the age of 5 were overweight or affected by obesity in 2020 [5]. As such, the global spread of obesity has been labeled a pandemic. The World Obesity Federation has estimated that 254 million children and adolescents will live with obesity in 2030 [6]. Moreover, childhood obesity is associated with cardiometabolic and psychosocial comorbidity, including atherosclerosis, hypertension, dyslipidemia, type 2 diabetes mellitus, as well as reduced overall survival [5].

It is well-known that the development of obesity results from both innate biological predisposition, environmental factors, and incorrect lifestyles (i.e., excessive dietary intake, sedentary habits), resulting in an imbalance between calorie consumption and energy expenditure [7]. Although changes in the environment have undoubtedly driven the steep increase over the last decades, a strong genetic component determines how people respond to an ‘obesogenic’ environment in terms of body weight, with an estimated heritability of 40–70%, as demonstrated by twin, family, and adoption studies, and the different efficacy of current therapies [8].

## 2. Methods

### Literature Search

This narrative review was developed to provide a clinically oriented overview of the genetic architecture, phenotypic presentation, diagnostic evaluation, and emerging precision therapies for genetic obesity in children and adolescents. A structured literature search was performed in PubMed/MEDLINE, Scopus, and Web of Science to identify relevant studies published from 2000 to 2025. Search terms included combinations of “childhood obesity”, “pediatric obesity”, “genetic obesity”, “monogenic obesity”, “syndromic obesity”, “polygenic obesity”, “leptin”, “melanocortin”, “MC4R”, “LEP”, “LEPR”, “POMC”, “PCSK1”, “genetic testing”, “polygenic risk score”, and “precision medicine”. Reference lists of relevant systematic reviews, consensus statements, and guidelines were additionally screened to identify pertinent publications not retrieved through the electronic search. Non-English articles, conference abstracts, and studies lacking genetic validation were excluded. Studies were selected based on their relevance to the scope of the review, with particular emphasis on studies addressing pediatric populations, genetic mechanisms of obesity, genotype–phenotype relationships, diagnostic approaches, and genetically informed therapeutic strategies. Selected studies conducted in adult populations were also considered when they provided essential evidence for the biological mechanisms discussed. No formal risk-of-bias assessment was performed because this article was designed as a narrative rather than a systematic review.

## 3. From Gene Discovery to Biological Pathways

There is a growing focus on genes implicated in obesity, and novel genes associated with childhood obesity continue to be reported [9,10,11]. As we will discuss in detail in the following paragraphs, genetic causes of obesity can be classified in different ways, including:

– monogenic obesity: a severe, early-onset form of obesity, caused by a single-gene mutation, with little or no influence of the environment [12]
– polygenic obesity: a common multifactorial form of obesity, resulting from an interaction between the obesogenic environment and genetic polymorphisms [13]
– syndromic obesity: in this condition, excessive weight is the predominant manifestation of a broader multisystem disorder [14].

Genetic discoveries in obesity have evolved in parallel with technological advances in genomic analysis. Early investigations focused primarily on families with severe obesity and relied on candidate-gene approaches guided by observations in animal models [15]. The first breakthrough in human obesity genetics followed the identification of congenital leptin deficiency in 1997, establishing the critical role of leptin signaling in energy homeostasis [16]. This study led to the identification of highly penetrant mutations affecting appetite regulation and energy homeostasis.

The introduction of high-throughput sequencing technologies dramatically expanded the ability to identify disease-causing variants. Whole exome sequencing (WES) and whole genome sequencing (WGS) now allow for the systematic investigation of coding and non-coding regions, facilitating the discovery of novel obesity genes and previously unrecognized biological pathways [17]. In contrast, the genetic basis of common obesity emerged through genome-wide association studies (GWAS), which revealed hundreds of loci associated with BMI and adiposity traits [18,19,20]. Although the individual contribution of most variants is modest, collectively, they account for a substantial proportion of inherited obesity susceptibility. The first GWAS for obesity traits was published in 2007 [21]. Since then, more than 1000 loci have been associated with BMI and obesity-related traits [22]. Importantly, findings from rare monogenic disorders and common polygenic obesity increasingly converge on shared biological networks, particularly those involved in the hypothalamic regulation of energy balance. Although many obesity-associated loci identified in adults also show associations with BMI during childhood and adolescence [23], the contribution of genetic variants is not uniform across the life course. Genetic effects may vary according to age, developmental stage, and sex, and the genetic correlation between childhood and adult adiposity is incomplete [24,25]. These observations suggest that the biological determinants of obesity are partly shared across the lifespan but are also influenced by age-specific mechanisms. Moreover, it was speculated that low-frequency (minor allele frequency, MAF 1–5%) and rare (MAF < 1%) variants would have larger effects than common variants and thus be easier to detect. Nevertheless, even large-scale studies, such as the one by Turcot et al., identified only a few robust associations for rare coding variants [26]. Despite the difficulties in validating causative mutations, tissue and functional analyses can provide insights into potential mechanisms.

### The Leptin–Melanocortin Pathway

Over the last three decades, human and animal genetic studies have identified the leptin–melanocortin system as the principal neuroendocrine network regulating food intake and body-weight homeostasis. Rather than acting as a simple satiety pathway, this system (that involves the hypothalamus, the pituitary gland, hippocampus, the insula and the substantia nigra) integrates peripheral signals of nutritional status with central neuronal circuits controlling hunger, reward, and energy expenditure [13,27].

Leptin, a hormone predominantly secreted by adipose tissue and encoded by *LEP*, functions as a signal of long-term energy stores. Circulating leptin concentrations generally reflect fat mass and fluctuate according to changes in energy availability (Figure 1). After reaching the hypothalamus, leptin binds to its receptor (LEPR) on neurons of the arcuate nucleus, where it stimulates anorexigenic pathways while suppressing orexigenic signaling. Activation of pro-opiomelanocortin (POMC)-expressing neurons promotes the production of melanocortin peptides through the action of prohormone convertases, particularly PCSK1. These peptides subsequently activate melanocortin receptors, especially MC4R, in downstream hypothalamic nuclei, resulting in reduced appetite and increased energy expenditure. Conversely, neurons expressing agouti-related peptide (AgRP) and neuropeptide Y (NPY) counteract melanocortin signaling and promote feeding behavior [28]. The physiological regulation of body weight depends on the dynamic balance between these opposing neuronal populations. Genetic defects affecting any component of this pathway—including *LEP, LEPR, POMC, PCSK1, MC4R, SH2B1, MRAP2, SIM1, ADCY3, BDNF*, and *NTRK2*—can disrupt appetite control and lead to severe early-onset obesity [29]. Importantly, clinical studies have demonstrated that the degree of impairment of melanocortin signaling correlates with hyperphagia severity, supporting the concept that this pathway operates along a functional continuum rather than through an all-or-none mechanism [30]. In addition to regulating food intake, it also regulates food preference, with individuals who carry mutations in *MC4R* showing a preference for food with a higher fat content [31].

## 4. Genetic Forms of Obesity

Classically, the genetics of obesity has two forms, i.e., syndromic and non-syndromic; the latter can be further distinguished in monogenic and polygenic.

### 4.1. Syndromic Obesity

In this condition, obesity is accompanied by variable combinations of neurodevelopmental impairment, congenital anomalies, endocrine dysfunction, sensory deficits, or characteristic dysmorphic features. Consequently, the clinical evaluation should extend beyond anthropometric measurements and include a careful assessment of developmental history, neurological findings, and extra-metabolic manifestations. Although the underlying molecular defects are highly heterogeneous, most syndromic forms can be grouped according to their predominant clinical presentation. Disorders primarily affecting hypothalamic development or function are typically characterized by severe hyperphagia and impaired satiety, whereas ciliopathies frequently combine obesity with retinal degeneration, renal abnormalities, and limb malformations. Other syndromes result from chromosomal rearrangements or epigenetic defects, producing more complex phenotypes. Over 80 different syndromes presenting with pediatric obesity have been described [32]. **Prader–Willi syndrome (PWS)** remains the most common syndromic cause of childhood obesity. The phenotype evolves considerably with age, beginning with neonatal hypotonia and poor feeding, followed by progressive hyperphagia and rapid weight gain during early childhood. The diagnosis should be suspected whenever severe obesity develops in association with hypotonia, developmental delay, hypogonadism, and characteristic behavioral disturbances. Since the disorder results from the loss of expression of paternally inherited genes within chromosome 15q11–q13, methylation analysis remains the diagnostic test of choice [33]. **Bardet–Biedl syndrome (BBS)**, in contrast, illustrates the multisystem consequences of primary ciliary dysfunction. Although obesity usually develops early in life, it is rarely an isolated finding. Progressive retinal dystrophy, postaxial polydactyly, renal involvement, hypogonadism, and varying degrees of cognitive impairment provide important diagnostic clues. The remarkable genetic heterogeneity of BBS reflects the central role of primary cilia in energy homeostasis and organ development [34]. **Alström syndrome** is similarly characterized by cone-rod dystrophy as key manifestation together with bilateral hearing loss and progressive deterioration of the kidneys. Other syndromic conditions generally present with milder obesity but distinctive associated features that may facilitate clinical recognition. **Fragile X syndrome,** due to the unbalanced growth of polymorphic CGG trinucleotide repeats present in the *FMR1* (fragile X mental retardation) gene, may be accompanied by increased body weight together with intellectual disability and behavioral disorders [35], whereas **Cohen syndrome** (caused by mutations in COH1 gene) combines truncal obesity with microcephaly, retinal dystrophy, neutropenia, and developmental delay [36]. Similarly, obesity associated with **pseudohypoparathyroidism type Ia** is usually accompanied by the characteristic skeletal features of Albright hereditary osteodystrophy (brachydactyly and widening of long bones) and resistance to multiple hormones due to pathogenic variants affecting the maternal *GNAS* allele [37]. Finally, in **WAGR syndrome** (Wilms tumor, anorexia, ambiguous genitalia, and mental retardation), obesity develops predominantly in individuals carrying deletions involving the *BDNF* locus, highlighting the importance of neurotrophic signaling in appetite regulation [38]. Other rare syndromes with childhood obesity include Carpenter syndrome, CHOPS syndrome, Chudley–Lowry syndrome, Laron syndrome, and Smith-Magenis syndrome [32].

### 4.2. Monogenic Obesity

Monogenic obesity involves single-gene mutations or chromosomal deletions. The subjects typically exhibit severe early-onset (<5 years old) obesity and rapid weight gain in the first 2 years associated with hyperphagia and sometimes endocrine disorders (e.g., adrenal insufficiency, hypogonadism) or immunodeficiency. Additionally, as monogenic obesity often demonstrates a recessive inheritance pattern, consanguinity in populations further increases the chance of identifying mutations [39]. These rare forms of childhood obesity are uncommon, accounting for up to 13% of children referred to a tertiary center for pediatric obesity [40]. As previously discussed, the leptin–melanocortin axis is the most common pathway involved in monogenic obesity, but other mechanisms could be involved, as listed in Table 1. ***MC4R*** deficiency is the most prevalent inherited cause of obesity, with an estimated prevalence of 2–5% among all obese children, with the c.496G>A variant in the *MC4R* gene being the most frequent in early-onset obese patients with hyperphagia [41]. To date, according to HGMD (http://www.hgmd.cf.ac.uk/ac, accessed on 10 June 2026), 213 total mutations are reported for this gene. Pathogenicity of *MC4R* mutations is more common in northern European populations than other ethnic groups [42]. Recently, it has been discovered that gain-of-function, but also loss-of-function mutations reduce BMI and cardiovascular risk, highlighting the importance of *MC4R* in the regulation of body weight and fat storage [43,44]. Genetic variants in the ***LEP*** **gene** are associated with very low levels of blood leptin, which on the contrary, are typically elevated in children with obesity, and strictly correlated with body fat mass. Its pathogenic variants have been found in nearly 70 patients worldwide. Patients usually present with severe hyperphagia, extreme childhood obesity beginning in the first year of life, and often also hypogonadism and hypothyroidism [45]. ***LEPR*** dysfunction can also cause a very similar clinical phenotype [46]; more than 60 different variants are already known. Other forms of monogenic obesity have distinctive features, such as red hair or pale skin associated with adrenal insufficiency and sometimes cholestasis for ***POMC*** compound heterozygous or homozygous pathogenic mutations [47] or persistent malabsorptive diarrhea and multiple endocrine impairments for loss-of-function ***PCSK1*** pathogenic mutations [48]. Moreover, growing literature is reporting rarer causes of monogenic obesity, including ***SH2B1*** mutation causing severe early-onset obesity either isolated or with developmental delay [49,50], **single minded-1 (*****SIM1*****)** syndrome with in Prader–Willi-like features [51,52], ***ADCY3*** [53], and **agouti signaling protein (*****ASIP*****)** [54].

**Table 1:** **Main Genetic Determinants of Monogenic Obesity in Children.** Genes are grouped into four biological pathways: the leptin–melanocortin axis, neurotrophic and synaptic signaling, ciliary and intracellular second-messenger pathways, and synaptic development and chromatin remodeling.

Gene	Full Name	Locus	Inheritance	Protein Function	Clinical Phenotype	Notes	Reference
Leptin –Melanocortin Pathway							
*LEP*	Leptin	7q31.3	AR	ADIPOKINE Adipose-secreted hormone; suppresses appetite and promotes energy expenditure via POMC activation	Early onset, severe obesity, hyperphagia Hypogonadotropic hypogonadism, recurrent infections, undetectable serum leptin	Responds to recombinant leptin therapy	[44]
*LEPR*	Leptin Receptor	1p31.3	AR	LEPTIN SIGNALING Hypothalamic receptor for leptin; activates JAK2–STAT3 pathway; regulates POMC and AgRP neuron activity	Early onset, severe obesity, hyperphagia Elevated serum leptin, pituitary dysfunction, short stature	Setmelanotide approved	[55]
*POMC*	Pro-opiomelanocortin	2p23.3	AR	NEUROPEPTIDE PRECURSOR Precursor of α-MSH, β-endorphin, ACTH; regulates HPA axis and pigmentation	Early onset, severe obesity, hyperphagia Red hair, pale skin, adrenal insufficiency (ACTH deficiency), normal-to-elevated leptin	Setmelanotide approved; skin/hair phenotype is a diagnostic clue	[55]
*PCSK1*	Proprotein Convertase Subtilisin/Kexin Type 1	5q15	AR	PROCESSING ENZYME Neuroendocrine serine protease; cleaves POMC → α-MSH and β-endorphin; also processes proinsulin, proGLP-1, and other prohormones	Early onset obesity, malabsorptive diarrhea Reactive hypoglycemia, hypogonadism, hypothyroidism, elevated proinsulin	Setmelanotide approved. Neonatal persistent diarrhea is a hallmark	[55]
*SH2B1*	SH2B Adaptor Protein 1	16p11.2	AD	ADAPTOR PROTEIN Enhances JAK2 activation downstream of LEPR and insulin receptor; regulates neuronal and peripheral energy sensing	Early onset, severe obesity, hyperphagia Insulin resistance, maladaptive behavior, developmental delay (in 16p11.2 deletion carriers)	16p11.2 deletion (encompassing SH2B1) linked to autism spectrum and obesity	[49]
*MC4R*	Melanocortin 4 Receptor	18q21.32	AD	MELANOCORTIN RECEPTOR G-protein coupled receptor in PVN; activated by α-MSH (anorexigenic) and antagonized by AgRP (orexigenic); regulates food intake and energy expenditure via cAMP	Early onset, severe obesity, hyperphagia Hyperinsulinemia, increased lean mass and linear growth, normal puberty;	Most common monogenic obesity gene	[42]
*MC3R*	Melanocortin 3 Receptor	20q13.2	AD	MELANOCORTIN RECEPTOR Expressed in the hypothalamus and limbic system; modulates energy partitioning, feed-forward regulation of the melanocortin axis, and circadian feeding rhythms	Childhood obesity, increased adiposity Reduced resting metabolic rate, elevated leptin levels, and altered insulin sensitivity; milder phenotype than MC4R	Loss-of-function MC3R variants linked to reduced childhood growth, delayed pubertal onset, and lower adult lean mass,	[56]
*AGRP*	Agouti-Related Protein	16q22.2	AR	OREXIGENIC NEUROPEPTIDE Endogenous inverse agonist/antagonist at MC3R and MC4R; expressed in ARC AgRP/NPY neurons; promotes food intake	Increased BMI, hyperphagia	Rare gain-of-function variants cause severe obesity	[57]
*ASIP*	Agouti Signaling Protein	20q11.22	AD	NEUROPEPTIDE Competitive antagonist of melanocortin peptides at MC1R	Early-onset obesity, overgrowth, red hair and hyperinsulinemia	Aberrant, ectopic ASIP expression leads to obesity in mice and humans	[54]
Neurotrophic & Synaptic Signaling							
*BDNF*	Brain-Derived Neurotrophic Factor	11p14.1	AD	NEUROTROPHIN Released by VMH neurons downstream of MC4R; binds NTRK2 (TrkB) to suppress appetite and increase energy expenditure; critical for hypothalamic circuit development	Early onset obesity, hyperphagia Cognitive impairment, hyperactivity, memory deficits	Associated with cognitive defects, epilepsy, obesity, tumorigenesis, neurodegenerative disorders and syndromes (e.g., WAGR syndrome when in 11p14.1 deletion)	[58]
*NTRK2*	Neurotrophic Receptor Tyrosine Kinase 2 (TrkB)	9q21.33	AD	TYROSINE KINASE RECEPTOR High-affinity receptor for BDNF; activates MAPK, PI3K and PLCγ pathways; regulates hypothalamic appetite circuits, memory and synaptic plasticity	Early onset, severe obesity, hyperphagia Intellectual disability, hyperactivity, stereotyped behaviors, impaired pain sensation	Rare dominant mutations (e.g., Y722C) cause extreme hyperphagia and obesity-intellectual disability syndrome	[59,60]
*SIM1*	Single-Minded Homologue 1	6q16.3	AD	TRANSCRIPTION FACTOR bHLH-PAS transcription factor essential for PVN development; regulates MC4R expression and oxytocin-producing neuron differentiation in hypothalamus	Early onset, severe obesity, hyperphagia Prader–Willi-like features (hypotonia, short stature, hypogonadism), intellectual disability in some cases	-	[52]
Ciliary Signaling & Intracellular Messengers							
*ADCY3*	Adenylyl Cyclase 3	2p23.3	AR	CAMP SYNTHESIS Generates cAMP at primary cilia of hypothalamic neurons downstream of MC4R and LEPR; regulates neuronal and adipocyte energy sensing	Early onset severe obesity, anosmia	Anosmia distinguishes from other MC4R-axis defects	[53]
*SEMA3*	Semaphorin-3 (family)	7q21.11 (SEMA3A)	AD	AXON GUIDANCE/CILIA Secreted semaphorins guide melanocortinergic axonal projections to PVN; regulate primary cilia length in hypothalamic neurons and modulate LEPR signal transduction	Childhood obesity, hyperphagia Anosmia/hyposmia (Kallmann-like), altered reproductive axis (SEMA3A and SEMA3E)	Rare heterozygous variants in SEMA3A, SEMA3C, SEMA3D identified in obese children; functional overlap with ciliopathy spectrum	[61]
Synaptic Development & Chromatin Remodeling							
*KSR2*	Kinase Suppressor of Ras 2	12q24.22	AD	SCAFFOLD PROTEIN Coordinates RAS–ERK and AMPK signaling; regulates fatty acid oxidation and glucose metabolism in peripheral tissues and hypothalamic neurons	Early onset obesity, severe hyperphagia Bradycardia, reduced basal metabolic rate, insulin resistance; responds to metformin	Metformin improves metabolic phenotype in KSR2 patients by bypassing AMPK defect	[62]
*MAGEL2*	MAGE Family Member L2	15q11.2	AD	UBIQUITIN LIGASE REGULATOR Imprinted gene (paternal expression); regulates LEPR surface expression; also modulates circadian clock and hypothalamic neuropeptide secretion	Severe obesity, hyperphagia, neonatal hypotonia	Schaaf–Yang syndrome when mutated, Prader–Willi syndrome when in paternal 15q11-q13 deletion)	[63]

**Abbreviations**: AD, autosomal dominant; AgRP, agouti-related peptide; AMPK, AMP-activated protein kinase; AR, autosomal recessive; ARC, arcuate nucleus; BMI, body mass index; *MC4R*, melanocortin-4 receptor; PVN, paraventricular nucleus; SNP, single nucleotide polymorphism; VMH, ventromedial hypothalamus; WAGR, Wilms tumor–Aniridia–Genitourinary anomalies–intellectual disability.

### 4.3. Polygenic Obesity

Polygenic obesity reflects the cumulative contribution of a large number of common genetic variants, each generally exerting a small effect on susceptibility to adiposity. These genetic effects operate within a broader biological and environmental context, which includes nutritional exposures, physical activity, socioeconomic factors, and developmental influences. Obesity has always been considered the prototype of polygenic diseases [64]. Individually, these genetic variants associated with weight gain confer modest effects; however, their cumulative burden contributes substantially to obesity risk. Many loci are enriched in genes expressed in the central nervous system, reinforcing the concept that obesity is primarily a neurobehavioral disorder of appetite regulation rather than solely a metabolic disease [11]. The genetic determinants of polygenic obesity in children are reported in Table 2. Among the obesity-related genetic variants, those located in the fat mass and obesity-associated (*FTO*) gene locus were the first ones that showed a strong association with BMI. The *FTO* gene, expressed in the hypothalamus, is located on chromosome 16 and encodes a demethylation enzyme implicated in the control of energy homeostasis, food intake, and energy expenditure [65]. Several studies have demonstrated that the variants rs9939609 and rs9930506 in the *FTO* gene may increase the risk of obesity in both adults and children [66]. Considering the critical pathogenic role of pro-inflammatory cytokines in obesity, further genetic association studies focused on the influence of variants in genes encoding for these molecules in obesity [67]. For example, the rs1800629 variant in the TNF-α gene, associated with increased expression of the cytokine in adipose tissue, was found to be more frequent in children with obesity than in lean subjects [68].

Notably, emerging evidence indicates that monogenic and polygenic obesity share overlapping biological pathways, and the distinction between them is increasingly recognized as a continuum rather than a dichotomous entity [69]. Rare highly penetrant mutations (causing severe early-onset obesity) and common regulatory variants (that contribute to population-level BMI variance) often affect the same genes or signaling networks, for example, the *ADCY3* and *MC4R* genes.

**Table 2:** Main genetic determinants of polygenic obesity in children.

Gene	Full Name	Locus	Protein Function	Clinical Phenotype	Notes	Ref.
*FTO*	Fat Mass and Obesity Associated (α-ketoglutarate dioxygenase)	16q12.2	N6-METHYLADENOSINE RNA DEMETHYLASE Regulates mRNA stability of metabolic transcripts; regulates IRX3/IRX5 expression in hypothalamus and adipocytes, controls thermogenesis and adipogenesis	Increased BMI, fat mass, food intake Effect detectable from age 2–3; no syndromic features	Largest effect-size common BMI locus; effect amplified by physical inactivity and high-energy diet Median change in BMI of ~0.4 kg/m^2^ per allele	[66]
*TNF-alpha*	Tumor necrosis factor -alpha	6p21.33	CYTOKINE Master regulator of immune responses; also triggers apoptotic cell death and regulates tissue regeneration, bone and lipid metabolism	Increased BMI especially during adolescence	Discordant effects on insulin resistance and metabolic syndrome	[68]
*MC4R*	Melanocortin 4 Receptor	18q21.32	MELANOCORTIN RECEPTOR See Table 1; common non-coding variants rank among the top GWAS signals for BMI and waist circumference, independent of rare coding mutations	BMI elevation, phenotypically milder than rare coding variants	Median change in BMI of ~0.2 kg/m^2^ per allele	[66]
*MC3R*	Melanocortin 3 Receptor	20q13.2	MELANOCORTIN RECEPTOR See Table 1	Childhood obesity Reduced resting metabolic rate, elevated leptin levels, and altered insulin sensitivity	Biallelic rare variants and common variants contribute to polygenic risk	[70]
*TMEM18*	Transmembrane Protein 18	2p25.3	NUCLEAR MEMBRANE PROTEIN Highly expressed in hypothalamus; regulates transcription of appetite and metabolic genes (unclear exact mechanism)	Childhood BMI increase, central adiposity	Effect size larger in children than adults; may act during critical developmental windows	[71]
*GNPDA2*	Glucosamine-6-Phosphate Deaminase 2	4p13	HEXOSAMINE PATHWAY Expressed in the hypothalamus, involved in hexosamine biosynthesis pathway, which acts as a cellular nutrient sensor; regulates insulin signaling	Increased BMI, no other distinct clinical features	Potential association with unhealthy diet	[72]
*NEGR1*	Neuronal Growth Regulator 1	1p31.1	CELL ADHESION/SYNAPTOGENESIS GPI-anchored cell adhesion molecule; promotes hypothalamic synaptogenesis and axonal connectivity; modulates serotonergic and melanocortinergic circuit formation in the developing brain	Increased BMI from childhood Potential association with depression and anxiety; no discrete syndromic phenotype	Consistently replicated across European and non-European pediatric GWAS	[73]
*TFAP2B*	Transcription Factor AP-2 Beta	6p12.3	TRANSCRIPTION FACTOR Regulates adipogenesis, lipid storage genes, and sympathetic nervous system differentiation; expressed in hypothalamus and adipose tissue	Increased BMI and fat mass	Rare germline mutations cause Char syndrome (patent ductus arteriosus, facial features)	[74]
*OLFM4*	Olfactomedin 4	13q14.3	SECRETED GLYCOPROTEIN Modulates Wnt/β-catenin and NF-κB signaling; expressed in gut and adipose tissue; proposed role in gut-derived satiety signaling and adipocyte differentiation	Increased childhood BMI Associated with altered gut microbiome composition	Emerging locus in pediatric GWAS meta-analyses	[75]
*ADCY3*	Adenylyl Cyclase 3	2p23.3	CAMP SYNTHESIS See Table 1	Severe obesity		[76]
*PCSK1*	Proprotein Convertase Subtilisin/Kexin Type 1	5q15	PROCESSING ENZYME See Table 1	Severe obesity	Variant p.N221D contributes polygenic risk	[77]
*AGRP*	Agouti-Related Protein	16q22.2	OREXIGENIC NEUROPEPTIDE See Table 1	Increased BMI, hyperphagia		[78]
*BDNF*	Brain-Derived Neurotrophic Factor	11p14.1	NEUROTROPHIN See Table 1	Early onset obesity, hyperphagia Cognitive impairment, hyperactivity, memory deficits		[79]

**Abbreviations:** BMI, body mass index; GWAS, genome-wide association study.

### 4.4. Epigenetics

Epigenetic mechanisms represent an additional regulatory layer that may contribute to the expression of obesity-related phenotypes and may mediate, at least in part, the effects of environmental exposures [80]. Epigenetic modifications such as histone modification, DNA methylation, micro-RNA and chromatin remodeling caused changes in the gene expression pattern, leading to the development of obesity. The rs10487505 variant in the long non-coding RNA LncOb has been associated with BMI in adults with obesity [81]. Moreover, obesity-related genes have been linked to gene–environment interactions and the epigenome; for instance, *MC4R* and *leptin* genes are also involved in altering methylation patterns due to a high fat diet [82]. In addition, pre-natal exposure to chemical obesogens (phthalates, parabens and other phenols) can strongly influence the subsequent predisposition to obesity [83]. Of note, these compounds (but also parental dietary habits) may control the processes of epigenetic transgenerational inheritance of adult-onset diseases by modulating DNA methylation and epimutations in reproductive cells [84,85]. Further studies using the analysis of methylation quantitative trait loci (meQTLs) with GWAS may help in understanding how genetic predisposition and environmental factors interact with each other to determine the methylation status that predisposes the development of an obese phenotype in children.

## 5. Diagnostic Approach to Suspected Genetic Obesity

The diagnostic evaluation of a child with severe obesity should begin with a fundamental clinical question: does this patient follow the typical trajectory of common obesity, or are there features suggesting an underlying genetic disorder? Although the vast majority of children have multifactorial obesity, early recognition of the minority with a monogenic or syndromic cause is essential because it may directly influence clinical management, genetic counseling, and in selected cases, therapeutic options.

Anthropometric measurements should always be interpreted within the overall clinical context. Recent studies have proposed BMI thresholds during the first years of life that may improve the selection of children for molecular testing. Kohlsdorf et al. suggested that BMI values > 27 kg/m^2^ or %BMIP95 > 140% at 2 years of age and BMI values > 33 kg/m^2^ or %BMIP95 > 184% at 5 years of age can be a valuable cut-off to identify children who should undergo genetic screening for monogenic obesity [86]. More recently, a European multicenter study identified a BMI ≥ 24 kg/m^2^ at 2 years of age as the optimal cutoff for differentiating patients with biallelic variants in the LEP, LEPR, and MC4R genes [87] However, BMI should be considered a screening tool rather than a diagnostic marker, since considerable overlap exists between genetic and multifactorial obesity [40].

The suspicion of genetic obesity should emerge from the combination of several findings. A simple diagnostic algorithm for the early identification of genetic obesity has been proposed (Figure 2). Age at onset represents one of the strongest indicators. Weight gain beginning during infancy or before five years of age, particularly when accompanied by persistent hyperphagia, should immediately raise clinical suspicion. Nevertheless, hyperphagia alone is insufficient, as eating behavior varies considerably among children with obesity. Subsequently, the identification of associated clinical manifestations that are unusual in common obesity, such as developmental delay, intellectual disability, congenital malformations, dysmorphic features (regarding facial shape, orientation of the palpebral slits, nose, philtrum, ears, head circumference, strabismus), neonatal hypotonia, or severe feeding difficulties during infancy, should prompt genetic screening. Similarly, a positive family history of severe early-onset obesity or, on the contrary, the absence of familial obesity background and parental consanguinity increases the likelihood of a monogenic etiology [88].

When suspecting genetic obesity, the second step is to assess endocrine (pituitary axes, serum leptin level, and phosphocalcium balance with vitamin D status) and metabolic characteristics (oral glucose tolerance test, lipid profile, liver enzymes), perform radiological imaging (X-rays of the hands, feet, and forearms in search of bone anomalies, brain MRI in case of intellectual disability and/or hypopituitarism), and an ophthalmological evaluation with an electroretinogram when visual problems emerge [89].

Once clinical suspicion has been established, the molecular diagnostic strategy should be individualized according to the phenotype. Children presenting with dysmorphic features, developmental disorders, or congenital anomalies may benefit from chromosomal microarray analysis or syndrome-specific investigations, including methylation testing for Prader–Willi syndrome or *FMR1* analysis when Fragile X syndrome is suspected. Conversely, isolated severe early-onset obesity with hyperphagia is more suggestive of defects affecting the leptin–melanocortin pathway and is appropriately investigated using targeted next-generation sequencing (NGS) panels that currently represent the most practical first-line approach. Trio (i.e., proband and parents) WES or WGS should be reserved at the moment for patients with a strong clinical suspicion despite negative first-line testing or for those with atypical phenotypes [90]. A useful web tool (ObsGen) developed in France helps clinicians in the diagnostic process when facing a suspicion of rare genetic obesity (https://obsgen.nutriomics.org).

Finally, molecular results should never be interpreted in isolation. Variant classification, segregation analysis, and detailed genotype–phenotype correlation remain essential for distinguishing pathogenic variants from variants of uncertain significance. Close collaboration among pediatric endocrinologists, clinical geneticists, molecular biologists, dietitians, psychologists, and other specialists therefore remains the cornerstone of an accurate diagnosis and appropriate long-term management, as is standard practice in many tertiary pediatric obesity centers [40].

## 6. From Genetic Diagnosis to Precision Medicine

The clinical relevance of establishing a molecular diagnosis extends beyond etiological clarification and labeling. Genetic characterization may directly influence therapeutic decisions, improve prognostic assessment, facilitate genetic counseling, and help patients and families understand the biological basis of the disease. What does a molecular diagnosis actually change in clinical practice?

- Psychosocial burden reduction. The diagnosis of genetic forms of obesity contests the common misconception that obesity is solely a result of lifestyle choices. This can have a significant psychological impact on patients living with obesity, who may be socially stigmatized as lacking self-discipline and esteem. A definitive diagnosis can help patients and families manage the load of uncertainty, reducing self and community blame. As such, based on the genetic diagnosis, the patients and their families can be oriented to patient associations, an essential support for disease acceptance and day-to-day life.
- Target treatment/management of obesity. After the diagnostic workup, a patient-tailored treatment plan should be designed by a trained multidisciplinary team and set up as soon as possible since an early intervention, even if only nutritional, is associated with better weight and metabolic outcome and could even prevent the development of obesity and other complications [40]. In cases of syndromic obesity, the patients should be evaluated for associated organ abnormalities or referred for disease-specific surveillance or therapy. For instance, patients diagnosed with BBS should be regularly seen by an ophthalmologist to determine the onset and degree of rod-cone dystrophy, screened for other visual defects, and monitored with renal function and blood pressure measurements every six months [91]. In PWS, the early start of growth hormone therapy can improve height, body composition, muscle strength, motor performance, and lipid metabolism [92]. The emergence of pathway-specific therapies has transformed the management of selected forms of genetic obesity. Subcutaneous injections of leptin in children and adults with *LEP* mutations resulted in weight loss and reduced food intake [93]. The second genotype-specific treatment for obesity is setmelanotide, a selective *MC4R* agonist approved by the FDA and EMA for BBS and *LEPR, PCSK1*, and *POMC* deficiency [94]. After a 1-year treatment with setmelanotide in phase III trials, the maximum weight loss was seen in patients with *POMC* deficiency (25.6% of their initial weight versus 12.5% in patients with *LEPR* deficiency), whereas the reduction in hunger was substantially larger in *LEPR*-deficient patients (−43.7% versus −27.1%) [95]. No specific treatment exists for syndromic excessive weight (except for setmelanotide in BBS), but it is of utmost importance that its general management (diet and physical activity) is adequate for age and for eventual associated eating disorder or cognitive impairment [14].
- Prognostic/risk stratification. As more variants are being discovered for common obesity, there is a growing expectation that genetic information will soon be used to identify individuals at risk of obesity. Knowing a person’s genetic susceptibility of gaining weight would allow for a more accurate prediction of who is at risk and for a more effective and prompter intervention. Khera et al., by incorporating the analysis from 2.1 million SNPs, proposed a polygenic risk score (PRS) that increased the prediction of BMI alteration up to 23% in adults and children [96]. Despite these strong associations with BMI and obesity, the predictive performance of the PRS is weak [97]. A pilot monogenic obesity risk score has been evaluated in a small population of children and adolescents with obesity, integrating clinical, behavioral, and metabolic features [98].

## 7. Discussion and Future Directions

The increasing availability of genomic technologies has substantially changed the way obesity can be conceptualized. GWAS have helped to statistically link genetic and phenotypic variation and have uncovered genetic variants that contribute cumulatively to human disease risk and complex trait variation. GWAS rely on precise phenotype measurements from hundreds of thousands of individuals. However, statistical power in child-focused GWAS is limited by smaller sample sizes compared to adult studies. Child cohorts are limited by ethical constraints, logistical hurdles in recruitment, and parental consent requirements. In addition, traits like BMI or internalizing symptoms often present with evolving definitions over time. When researchers meta-analyze child measurements across varying ages, this measurement burden amplifies noise, resulting in reduced statistical power [99]. To overcome this issue, Burrows et al. proposed a framework using BMI data from more than 70,000 children to identify age-varying genetic effects in GWAS [100]. Genetic obesity was initially regarded as a collection of rare disorders, often defined by highly penetrant mutations and distinctive phenotypes. More recent evidence, however, supports a broader continuum extending from rare monogenic and syndromic forms to common polygenic susceptibility, with substantial variation in the contribution of genetic factors across individuals and across the life course. Common obesity is influenced by the cumulative effect of thousands of genetic variants, most of which individually have very small effects, together with environmental and developmental determinants. At the other end of the spectrum, rare pathogenic variants may have much larger effects on appetite regulation and energy homeostasis. Thus, rather than representing mutually exclusive entities, monogenic and polygenic obesity can be viewed as points along a biological continuum. From a clinical perspective, this continuum is more relevant than a strict categorical distinction, because the same degree of obesity may result from markedly different combinations of genetic susceptibility, developmental factors, and environmental exposures.

Monogenic obesity remains particularly important in children because highly penetrant variants can lead to severe obesity and hyperphagia at a very early age, and in selected cases, may have direct therapeutic implications. Nevertheless, the phenotype is not always sufficiently distinctive to allow a molecular diagnosis on clinical grounds alone. Conversely, syndromic obesity may be suspected because of developmental abnormalities, congenital features, endocrine dysfunction, or sensory impairment rather than the severity of obesity itself. An additional challenge is the incomplete correspondence between genotype and phenotype. Even within the same genetic disorder, penetrance and expressivity may vary substantially, resulting in differences in age at onset, degree of hyperphagia, severity of obesity and BMI trajectories, and associated metabolic complications, reflecting residual receptor activity, the functional consequences of specific variants, genetic background, sex, age, and/or environmental exposures [101]. This variability is particularly relevant for *MC4R* deficiency, in which clinical manifestations may range from relatively mild obesity to severe early-onset phenotypes, and hyperinsulinemia and hyperphagia tend to diminish over time in an age-dependent fashion, with the underlying mechanisms remaining unclear [102]. Such observations argue against using a single anthropometric threshold as a proxy for genetic diagnosis and instead support an integrated assessment of age at onset, family history, eating behavior, associated features, and clinical trajectory. This phenotypic heterogeneity challenges the traditional approach of considering genetic obesity as synonymous with extreme BMI or early-onset obesity alone. This also means that molecular diagnosis should not replace phenotypic assessment. Instead, genotype and phenotype should be interpreted reciprocally: clinical characteristics can guide genetic testing and variant interpretation, while a molecular diagnosis can subsequently refine expectations regarding disease trajectory and associated manifestations. This approach is especially important for variants whose pathogenicity or clinical significance remains uncertain, and which may require re-evaluation over time [103].

Moreover, the genetic architecture of obesity is dynamic across the life course. This observation is particularly relevant to pediatric obesity. Childhood is characterized by major changes in adipose tissue distribution, appetite regulation, neuroendocrine development, growth, and pubertal maturation. Genetic variants may therefore exert different effects depending on the biological context in which they operate. Recent longitudinal analyses further support the presence of genotype-by-age interactions and incomplete genetic correlations between BMI measured at different developmental stages [104]. Accordingly, genetic susceptibility identified in childhood should not simply be interpreted as a fixed prediction of adult obesity. Instead, genetic information may contribute to understanding an individual’s trajectory while remaining dependent on age, sex, ancestry, and environmental exposures. Recognition of a biological contribution to obesity may help counter the persistent perception that excessive weight is primarily the consequence of individual behavior. Nevertheless, genetic information should be communicated carefully, emphasizing that genetic susceptibility does not imply inevitability and that environmental and behavioral factors remain relevant even in individuals with highly penetrant variants. This aspect is important when communicating genetic risk to families and when considering the potential clinical application of the PRS.

The PRS represents an attractive extension of obesity genetics because it quantifies the cumulative contribution of multiple common variants rather than focusing on a single pathogenic mutation. In pediatric populations, the PRS has been associated with BMI trajectories and other adiposity-related phenotypes, supporting the concept that inherited susceptibility can be detected before the full clinical phenotype becomes established, paving the road for tailored treatment and prevention. For example, Fang et al. found that children with a high BMI PRS have a lower risk of obesity when adhering to a healthy lifestyle [105]. However, the translation of these findings into routine clinical practice remains challenging [106,107]. Several limitations should be considered. First, predictive performance depends strongly on the ancestry composition of the population in which the score was developed and applied. For example, in the GIANT consortium meta-analysis of GWAS for BMI and height [108], all approximately 700,000 investigated participants were of European ancestry, raising concerns about their applicability in other ancestry groups and the potential consequences for health disparities. Second, a PRS captures only part of the genetic contribution to obesity and generally does not account adequately for rare variants, structural variation, or dynamic gene–environment relationships. Third, even when statistically significant at a population level, the ability of a PRS to improve individual clinical decision-making beyond conventional risk factors remains uncertain. Finally, the issue of communicating a PRS for obesity to patients remains relevant and should be addressed by future shared guidelines. A recent review of obesity PRS emphasized the need for further validation in large prospective cohorts before these tools can be routinely incorporated into clinical practice [107]. Therefore, the PRS should currently be regarded primarily as a research tool for population screening rather than a stand-alone diagnostic instrument.

Taken together, current evidence suggests that the most clinically useful model is not one in which genetic obesity is defined solely by the presence or absence of a pathogenic variant. Instead, genetic susceptibility should be interpreted as one component of a dynamic system involving rare and common variants, developmental stage, biological sex, ancestry, environmental exposures, and individual phenotype.

This integrated approach also changes the role of genetic testing. The relevant clinical question is not simply whether a child is “genetic” or “non-genetic”, but whether the available genetic information can explain relevant aspects of the phenotype and modify clinical management. In this context, age at onset, growth trajectory, hyperphagia, family history, developmental characteristics, and associated endocrine or syndromic manifestations remain essential components of the diagnostic process.

Future studies should therefore move beyond gene discovery toward longitudinal genotype–phenotype studies in well-characterized pediatric cohorts. Such research should integrate genomic, transcriptomic, epigenetic, metabolic, and environmental information and should evaluate whether genetically informed stratification improves prevention or treatment outcomes. Importantly, greater representation of diverse ancestral populations will be required to ensure that emerging genomic tools do not widen existing health disparities.

## 8. Limitations

This review has several limitations. First, it was designed as a narrative rather than a systematic review and therefore did not aim to provide a quantitative synthesis of the available evidence or a formal assessment of risk of bias. Although a structured literature search was performed, study selection was guided primarily by relevance to the clinical and biological scope of the review. Second, the rapidly evolving field of obesity genetics makes it difficult to provide an exhaustive account of all obesity-associated genes and variants. Third, evidence supporting several genetic associations derives predominantly from adult populations, and their relevance to pediatric obesity may not always be directly transferable. Fourth, current GWAS and PRS have an ancestry bias: they are derived predominantly from Western populations with limited representativeness of ancestry diversity. Newer generation population biobanks, such as the All of Us program [109], are designed to prioritize diversity and reflect the genetic makeup of the population, promising to further close this knowledge gap. Finally, genotype–phenotype relationships are influenced by age, sex, ancestry, environmental exposures, and ascertainment strategies, limiting the generalizability of findings across populations.

## 9. Conclusions

Genetic forms of childhood obesity range from rare monogenic disorders to common polygenic susceptibility. Advances in genetics and genomics are contributing to a deeper understanding of childhood obesity, facilitating the development of target therapeutic agents and challenging the traditional binary paradigm by supporting a dynamic model involving rare and common genetic variants. Functional genomics, epigenetic profiling, and integrative multi-omics approaches will be essential to translate genetic findings into biological insight. In children, the early identification of high-risk phenotypes offers a unique opportunity for preventive strategies before long-standing metabolic damage occurs. Moving forward, integrating genetic knowledge into routine care represents a critical step toward precision medicine in childhood obesity, which will require the integration of genetic diagnosis, behavioral interventions, pharmacotherapy, and long-term multidisciplinary care.

## Figures and Tables

**Figure 1:**
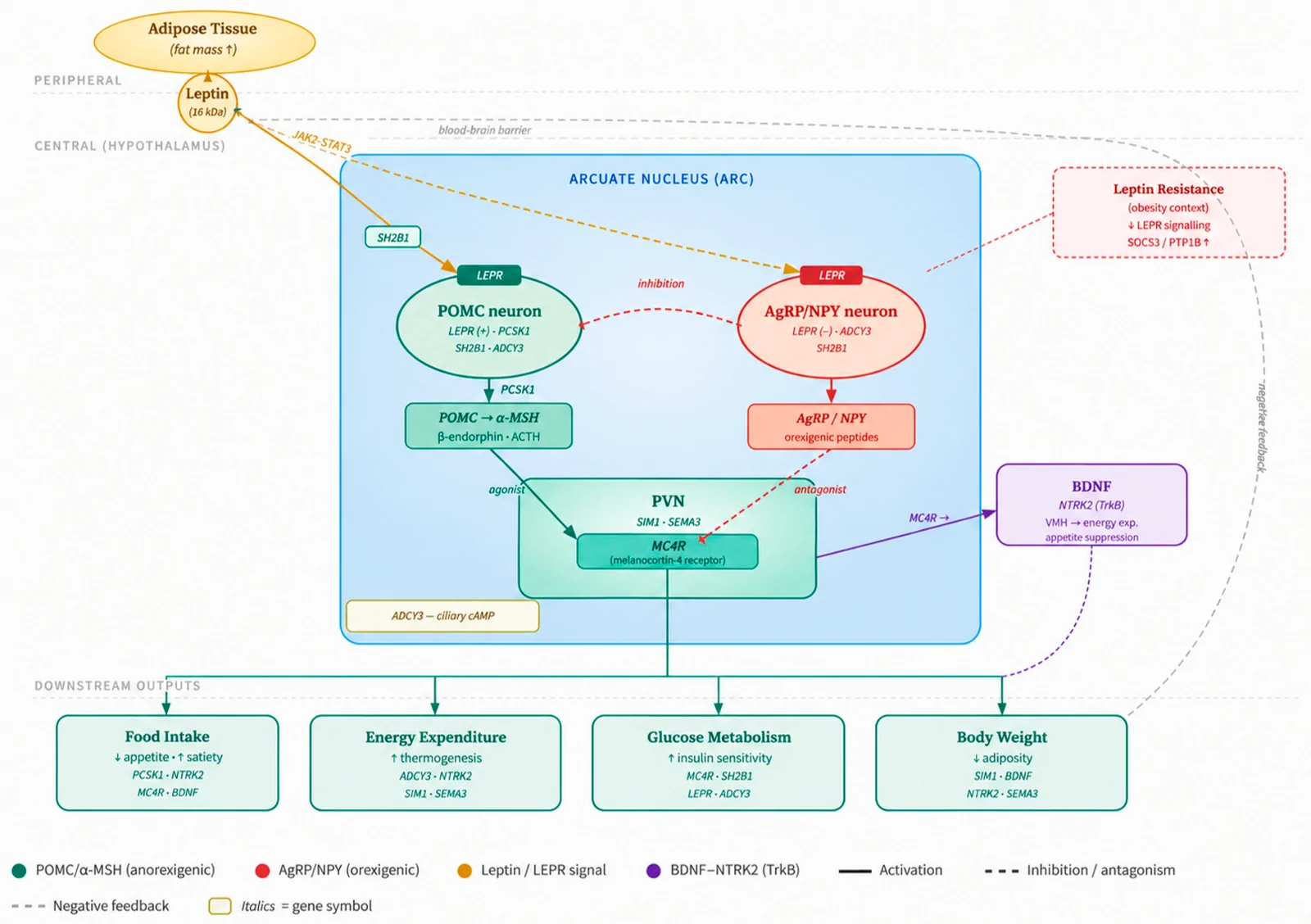
**The Leptin–Melanocortin Pathway.** Adipose-derived leptin circulates and crosses the blood–brain barrier to bind the leptin receptor (*LEPR*) on two antagonistic neuronal populations of the arcuate nucleus (ARC). **POMC neurons**: *LEPR* activation (JAK2–STAT3; potentiated by *SH2B1*) upregulates *POMC* transcription; the prohormone convertase *PCSK1* cleaves POMC to generate α-MSH, which acts as an MC4R agonist. Ciliary adenylyl cyclase *ADCY3* amplifies cAMP signaling in both POMC and AgRP neurons. **AgRP/NPY neurons**: inhibited by leptin; when disinhibited, they release AgRP (MC4R inverse agonist/antagonist) and NPY, driving hyperphagia. **PVN**: the transcription factor *SIM1* is required for PVN development and sustained MC4R expression; *SEMA3* (semaphorin-3) guides melanocortinergic axonal projections to the PVN. **MC4R** activation induces *BDNF* release in the ventromedial hypothalamus (VMH); BDNF signals via its receptor *NTRK2* (TrkB) to further suppress appetite and increase energy expenditure. Downstream outputs include reduced food intake, enhanced thermogenesis, improved insulin sensitivity, and decreased body weight. In obesity, leptin resistance (SOCS3/PTP1B-mediated) attenuates *LEPR* signaling, impairing pathway efficacy. A negative-feedback loop returns adiposity signals to the hypothalamus. **Gene symbols (italicized):** *LEPR*, leptin receptor; *PCSK1*, proprotein convertase subtilisin/kexin type 1; *SH2B1*, SH2B adaptor protein 1; *SIM1*, single-minded homologue 1; *SEMA3*, semaphorin-3; *ADCY3*, adenylyl cyclase 3; *NTRK2*, neurotrophic receptor tyrosine kinase 2; *BDNF*, brain-derived neurotrophic factor. ARC, arcuate nucleus; PVN, paraventricular nucleus; VMH, ventromedial hypothalamus; MC4R, melanocortin-4 receptor; α-MSH, α-melanocyte-stimulating hormone; AgRP, agouti-related peptide; NPY, neuropeptide Y. Generated using Claude 3.5 Sonnet (Anthropic, 2026).

**Figure 2:**
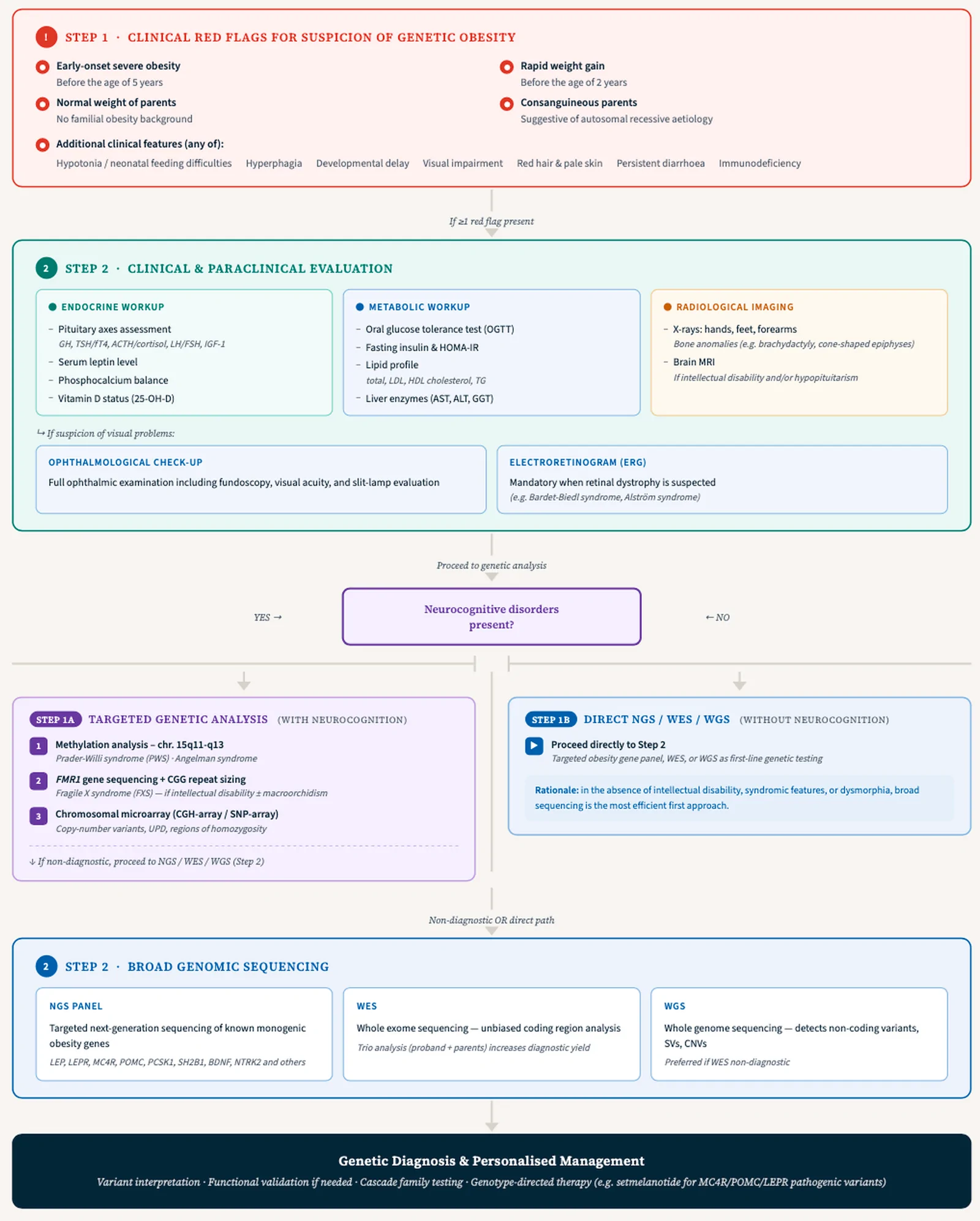
Diagnostic Flowchart for Genetic Obesity in Children. **Abbreviations**: CGH, comparative genomic hybridization; CNV, copy-number variant; ERG, electroretinogram; *FMR1*, Fragile X mental retardation 1; GH, growth hormone; HOMA-IR, homeostatic model assessment of insulin resistance; MRI, magnetic resonance imaging; NGS, next-generation sequencing; OGTT, oral glucose tolerance test; PWS, Prader–Willi syndrome; SNP, single nucleotide polymorphism; SV, structural variant; WES, whole exome sequencing; WGS, whole genome sequencing. Generated using Claude 3.5 Sonnet (Anthropic, 2026).

## Data Availability

The original contributions presented in this study are included in the article. Further inquiries can be directed to the corresponding author.

## Abbreviations

The following abbreviations are used in this manuscript:

BBS	Bardet–Biedl syndrome
BMI	Body mass index
FTO	Fat mass and obesity-associated
GWAS	Genome-wide association studies
MAF	Minor allele frequency
NGS	Next-generation sequencing
POMC	Pro-opiomelanocortin
PRS	Polygenic risk score
PWS	Prader–Willi syndrome
SNP	Single nucleotide polymorphism
WES	Whole exome sequencing
WGS	Whole genome sequencing
WHO	World Health Organization
